# Serum amyloid A1 mediates myotube atrophy via Toll‐like receptors

**DOI:** 10.1002/jcsm.12491

**Published:** 2019-08-23

**Authors:** Alexander Hahn, Melanie Kny, Cristina Pablo‐Tortola, Mihail Todiras, Michael Willenbrock, Sibylle Schmidt, Katrin Schmoeckel, Ilka Jorde, Marcel Nowak, Ernst Jarosch, Thomas Sommer, Barbara M. Bröker, Stephan B. Felix, Claus Scheidereit, Steffen Weber‐Carstens, Christian Butter, Friedrich C. Luft, Jens Fielitz

**Affiliations:** ^1^ Experimental and Clinical Research Center, Charité‐Universitätsmedizin Berlin Max Delbrück Center for Molecular Medicine in the Helmholtz Association Berlin Germany; ^2^ Cardiovascular hormones Max Delbrück Center for Molecular Medicine in the Helmholtz Association Berlin Germany; ^3^ Nicolae Testemiţanu State University of Medicine and Pharmacy Chișinău Moldova; ^4^ Signal Transduction in Tumor Cells Max Delbrück Center for Molecular Medicine in the Helmholtz Association Berlin Germany; ^5^ Department of Immunology, Institute of Immunology and Transfusion Medicine University Medicine Greifswald Germany; ^6^ Intracellular Proteolysis Max Delbrück Center for Molecular Medicine in the Helmholtz Association Berlin Germany; ^7^ Institute of Biology Humboldt‐University Berlin Berlin Germany; ^8^ DZHK (German Center for Cardiovascular Research), Partner Site Berlin Berlin Germany; ^9^ Department of Internal Medicine B, Cardiology University Medicine Greifswald Greifswald Germany; ^10^ DZHK (German Center for Cardiovascular Research), Partner Site Greifswald Greifswald Germany; ^11^ Department of Anesthesiology and Intensive Care Medicine, Campus Virchow‐Klinikum and Campus Charité Mitte Charité‐Universitätsmedizin Berlin Berlin Germany; ^12^ Berlin Institute of Health (BIH) Berlin Germany; ^13^ Department of Cardiology Heart Center Brandenburg and Medical University Brandenburg (MHB) Bernau Germany

**Keywords:** Sepsis, NF‐κB, CLP, Muscle atrophy, ICUAW

## Abstract

**Background:**

Critically ill patients frequently develop muscle atrophy and weakness in the intensive‐care‐unit setting [intensive care unit‐acquired weakness (ICUAW)]. Sepsis, systemic inflammation, and acute‐phase response are major risk factors. We reported earlier that the acute‐phase protein serum amyloid A1 (SAA1) is increased and accumulates in muscle of ICUAW patients, but its relevance was unknown. Our objectives were to identify SAA1 receptors and their downstream signalling pathways in myocytes and skeletal muscle and to investigate the role of SAA1 in inflammation‐induced muscle atrophy.

**Methods:**

We performed cell‐based *in vitro* and animal *in vivo* experiments. The atrophic effect of SAA1 on differentiated C2C12 myotubes was investigated by analysing gene expression, protein content, and the atrophy phenotype. We used the cecal ligation and puncture model to induce polymicrobial sepsis in wild type mice, which were treated with the IкB kinase inhibitor Bristol‐Myers Squibb (BMS)‐345541 or vehicle. Morphological and molecular analyses were used to investigate the phenotype of inflammation‐induced muscle atrophy and the effects of BMS‐345541 treatment.

**Results:**

The SAA1 receptors *Tlr2*, *Tlr4*, *Cd36*, *P2rx7*, *Vimp*, and *Scarb1* were all expressed in myocytes and skeletal muscle. Treatment of differentiated C2C12 myotubes with recombinant SAA1 caused myotube atrophy and increased *interleukin 6* (*Il6*) gene expression. These effects were mediated by Toll‐like receptors (TLR) 2 and 4. SAA1 increased the phosphorylation and activity of the transcription factor nuclear factor ‘kappa‐light‐chain‐enhancer' of activated B‐cells (NF‐κB) p65 via TLR2 and TLR4 leading to an increased binding of NF‐κB to NF‐κB response elements in the promoter region of its target genes resulting in an increased expression of NF‐κB target genes. In polymicrobial sepsis, skeletal muscle mass, tissue morphology, gene expression, and protein content were associated with the atrophy response. Inhibition of NF‐κB signalling by BMS‐345541 increased survival (28.6% vs. 91.7%, *P* < 0.01). BMS‐345541 diminished inflammation‐induced atrophy as shown by a reduced weight loss of the gastrocnemius/plantaris (vehicle: −21.2% and BMS‐345541: −10.4%; *P* < 0.05), tibialis anterior (vehicle: −22.7% and BMS‐345541: −17.1%; *P* < 0.05) and soleus (vehicle: −21.1% and BMS‐345541: −11.3%; *P* < 0.05) in septic mice. Analysis of the fiber type specific myocyte cross‐sectional area showed that BMS‐345541 reduced inflammation‐induced atrophy of slow/type I and fast/type II myofibers compared with vehicle‐treated septic mice. BMS‐345541 reversed the inflammation‐induced atrophy program as indicated by a reduced expression of the atrogenes *Trim63*/MuRF1, *Fbxo32*/Atrogin1, and *Fbxo30*/MuSA1.

**Conclusions:**

SAA1 activates the TLR2/TLR4//NF‐κB p65 signalling pathway to cause myocyte atrophy. Systemic inhibition of the NF‐κB pathway reduced muscle atrophy and increased survival of septic mice. The SAA1/TLR2/TLR4//NF‐κB p65 atrophy pathway could have utility in combatting ICUAW.

## Introduction

Critically ill intensive care unit (ICU) patients often develop muscle atrophy and weakness [ICU‐acquired weakness (ICUAW)] with increased morbidity and mortality.^1^, ^2^ The incidence of ICUAW is up to 90% in severe sepsis patients.^3^ Sequels persists for up to 5 years after discharge, underscoring substantial morbidity.^4^ Additionally, ICUAW is associated with high costs for the health care system.^4^, ^5^ Inflammation and sepsis clearly increase the risk for ICUAW, and inflammatory cytokines are associated with the incidence of ICUAW.^6^, ^7^ Skeletal muscle and skeletal myocytes are targeted by inflammatory cytokines, such as interleukin 1β (IL1β) and IL6,^8^, ^9^, ^10^ causing myocyte atrophy.^8^, ^11^, ^12^ Our attention was drawn to serum amyloid A1 (SAA1) that is associated with inflammation and acute‐phase responses during sepsis.^11^, ^13^While the main source of SAA1 is the liver,^13^ IL6 increases SAA1 expression and secretion in muscle.^8^, ^11^, ^14^ We earlier showed that SAA1 expression is greatly increased in ICUAW and that SAA1 accumulates in the myocyte membrane and in muscular interstitium.^11^ These findings were also observed in other studies.^15^, ^16^, ^17^, ^18^, ^19^ Nonetheless, a role of SAA1 as a perpetrator rather than a mere biomarker in ICUAW is imperfectly defined.^20^

SAA1 is produced by various tumours^21^, ^22^, ^23^ and is also associated with muscle wasting in cancer cachexia in mice.^24^ SAA1 cooperates with IL6 to mediate angiotensin II‐induced muscle atrophy,^9^ which is important for muscle wasting in heart failure patients. SAA1 receptors have been described in immune cells, including the purinergic receptor P2X7 (P2RX7), the CD36 receptor (CD36), the scavenger receptor class b member 1 (SCARB1), the VCP interacting membrane selenoprotein (VIMP), and Toll‐like receptors (TLR) 2 and 4.^25^, ^26^, ^27^, ^28^, ^29^, ^30^, ^31^ SAA1 activates the NLRP3 inflammasome via P2RX7 and thereby promotes IL1β activation and release from mouse and human macrophages.^25^ SAA1 also increases *IL6* and *TNF* expression by activating the CD36 receptor in rat macrophages and human embryonic kidney cells.^26^ SCARB1 mediates internalization of SAA in hepatocytes, and VIMP acts as an SAA1‐receptor in a mouse model of type 2 diabetes.^27^, ^28^ In mouse macrophages, SAA1 was shown to increase pro‐inflammatory cytokines, such as IL23α and tumor necrosis factor (TNF) via TLR2, and to increase nitric oxide via TLR4.^30^, ^31^, ^32^ Importantly, SAA1 increases *IL6* expression via TLR2 and TLR4 and activates the nuclear factor ‘kappa‐light‐chain‐enhancer' of activated B‐cells (NF‐κB) signalling pathway in dermal fibroblasts.^33^ However, whether or not the TLR2/TLR4/NF‐κB signalling pathway plays a role in SAA1‐induced myocyte atrophy *in vitro* and sepsis‐induced muscle atrophy *in vivo* is not known. We demonstrate that SAA1 acts on myocytes to cause atrophy by the TLR2/TLR4/NF‐κB pathway. We also show that inhibition of NF‐κB by Bristol‐Myers Squibb (BMS)‐345541 increases survival and diminishes skeletal muscle atrophy in septic mice. We suggest that SAA1 via activation of the TLR2/TLR4/NF‐κB atrophy pathway is mechanistically important in ICUAW.

## Methods

### Patient samples

The institutional ethics committee of the Charité approved the study; it has therefore been performed in accordance with the ethical standards laid down in the 1964 Declaration of Helsinki and its later amendments. Written informed consent was obtained from legal proxy (ICU patients), or the patients themselves (Charité EA2/061/06; http://www.controlled-trials.com, ISRCTN77569430). Clinical data were reported previously.^11^ Biopsy specimens were obtained at Day 15 after ICU admission; control samples were acquired from patients undergoing elective orthopaedic surgery as described elsewhere.^11^, ^34^, ^35^

### Animal model

The *Landesamt für Gesundheit und Soziales*, Berlin, Germany, approved the animal studies (G207/13, G129/12). Cecal ligation and puncture (CLP) surgery was performed to induce polymicrobial sepsis in 18‐week‐old male C57BL/6J mice as recently described.^11^, ^36^, ^37^ Sham mice were treated identically except for CLP (Supporting Information).

### Molecular and cell biology analysis

Routine techniques were performed as previously shown^11^, ^12^, ^35^, ^37^, ^38^, ^39^, ^40^ and are outlined (Supporting Information).

#### Cell culture experiments with BMS‐345541

We used BMS‐345541 to investigate if NF‐κB inhibition prevents sepsis‐induced muscle atrophy in the CLP mouse model *in vivo*.^41^ BMS‐345541 (4(2'‐aminoethyl)amino‐1,8‐dimethylimidazo(1,2‐a)quinoxaline; CAS‐number: 547757‐23‐3) is a highly selective cell permeable IκB kinase (IKK) 2 and IKK‐1 inhibitor, which blocks NF‐κB‐dependent transcription; it was produced by BMS and purchased from abcam (ab 144822). BMS‐345541 was previously shown to reduce CLP and LPS‐induced lung injury,^42^, ^43^ to reduce inflammation in rats with spinal cord injury, and to suppress inflammation in a model of cardiac graft rejection.^44^, ^45^ Due to its high bioavailability^41^ and its anti‐inflammatory characteristics without impairing immune cell activation^42^ and bacterial clearance, BMS‐345541 was well suited for our studies. A 2.5 mM stock solution of BMS‐345541 dissolved in 10% DMSO was prepared for all cell culture experiments. For short‐term treatment, a final concentration of 25 μM BMS‐345541 was used because Burke *et al*.^41^ showed that this concentration abolished lipopolysaccharide (LPS)‐induced production of TNF, IL1β, IL8, and IL6 in THP‐1 cells, a human leukemic monocyte cell line. Likewise, 25 μM BMS‐345541 abolished TNF‐stimulated IκBα‐phosphorylation.^41^ For long‐term treatment of myocytes (up to 72 h), we used 5 μM BMS‐345541. This concentration was chosen because the NF‐κB pathway is vital for cell function, and its complete inhibition over 72 h provokes toxic side effects. However, 5 μM BMS‐345541 was shown to completely inhibit IKK‐2, reduce IKK‐1 activity by approximately 50%, and substantially decrease LPS‐induced production of TNF, IL 1β, IL‐8, and IL‐6 in THP 1 cells^41^; 5 μM BMS‐345541 was shown to inhibit IκBα‐phosphorylation in the prostate cancer cell line PC‐3.^46^

#### Anti‐MuRF1 antibody

We generated a MuRF1 specific antibody against the C‐terminal *Mus musculus* MuRF1 cDNA fragment (amino acids 185‐355). The specificity of the antibody was tested by immunoblotting tissue lysates of tibialis anterior from wild type, *Trim63*/MuRF1 mutant and *Trim54*/MuRF3//*Trim 63*/MuRF1 double mutant mice and of lysates from C2C12 cells following siRNA mediated knockdown of MuRF1. For more information about the antibody please refer to Nowak *et al*.^47^

### Statistical tests

All experiments were performed independently and at least three times using biological triplicates each. The Mann–Whitney or two‐sided Student's *t*‐test was used to compare quantitative RT‐PCR gene expression data from mouse and cell culture samples. Distribution of myotube diameter of C2C12 myotubes were analysed by the two‐sided Student's *t*‐test. Survival curves were compared with a Mantel–Cox test. Differences were considered statistically significant at *P* < 0.05. Data are shown as mean ± standard error of the mean (SEM) in bar plots. GraphPad Prism® program (GraphPad software, version 6.0), Adobe Illustrator, version 16.0.0, and Photoshop, version 13.0, were used for plots and statistical calculations.

## Results

### Serum amyloid A1, Toll‐like receptors, and NF‐κB

**Figure 1:**
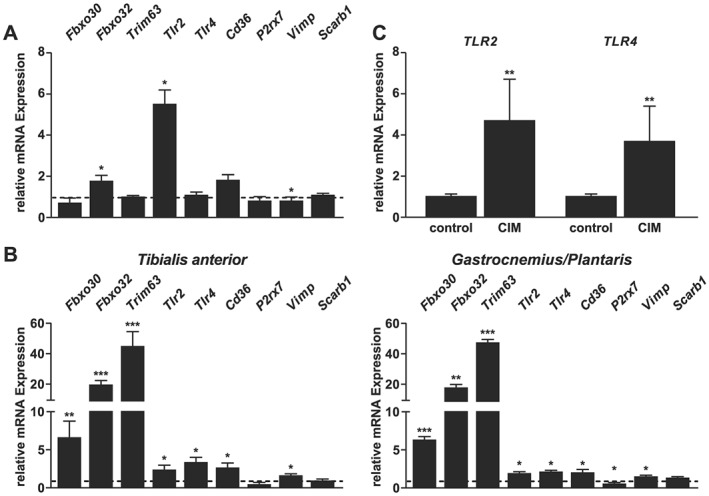
Expression of SAA1 receptors in C2C12 myotubes and skeletal muscle. (A) C2C12 myocytes were differentiated for 5 days and treated for 1 h with human recombinant SAA1 (20 μg/mL). qRT‐PCR analysis of SAA1 receptors *Tlr2*, *Tlr4*, *Cd36*, *P2rx7*, *Vimp*, *Scarb1*, and the atrogenes *Fbxo30*, *Fbxo32*, and *Trim63*. mRNA expression was normalized to *Gapdh*. Values of vehicle treated cells were set to 1 and are indicated as dotted line. *Cd36* indicates *cluster of differentiation 36*; *Fbxo30*, *F‐box protein 30*; *Fbxo32*, *F‐box protein 32*; *P2rx7*, *purinergic receptor P2X7*; *Scarb1*, *scavenger receptor class b member 1*; *Tlr2* indicates *Toll‐like receptor 2*; *Trim63*, *tripartite motif containing 63*; *Vimp*, *VCP interacting membrane selenoprotein*. (B) Twelve‐week‐old male WT mice were subjected to Sham (*n* = 3) or CLP (*n* = 5) surgery for 24 h. Expression of the SAA1 receptors *Tlr2*, *Tlr4*, *Cd36*, *P2rx7*, *Vimp*, and *Scarb1* and the atrogenes *Fbxo30*, *Fbxo32*, and *Trim63* was quantitated by qRT‐PCR in tibialis anterior and gastrocnemius/plantaris. mRNA expression was normalized to stably expressed *Gapdh*. Gene expression of sham treated mice was set to 1 and is indicated as dotted line. (C) qRT‐PCR analyses of *TLR2* and *TLR4* expression in control (no ICU subjects; *n* = 5) and CIM (15 days after ICU admission; *n* = 5) patients. *GAPDH* expression was used as a reference. Data are presented as mean ± SEM. *P ≤ 0.05, **P ≤ 0.01, and ***P ≤ 0.001.

We quantitated the expression of *Tlr2*, *Tlr4*, *Cd36*, *P2rx7*, *Vimp*, and *Scarb1* in differentiated C2C12 myotubes, and tibialis anterior and gastrocnemius/plantaris by quantitative real‐time polymerase chain reaction (qRT‐PCR). All SAA1 receptors investigated were expressed in myocytes and skeletal muscle (*Figure* 1A and 1B). We treated differentiated C2C12 myotubes with recombinant SAA1 and vehicle respectively and quantitated the expression of those SAA1 receptors by qRT‐PCR. We found an upregulation of *Tlr2* (5.4‐fold, *P* < 0.05) and a downregulation of *Vimp* (0.9‐fold, *P* < 0.05), whereas the expression of *Tlr4*, *Cd36*, *P2rx7*, and *Scarb1* remained unchanged (*Figure* 1A).

To investigate if SAA1 causes atrophy by directly increasing the expression of *F‐box only protein 30* (*Fbxo30*; encoding muscle ubiquitin ligase of the SCF complex in atrophy‐1, MuSA1), *Fbxo32* (encoding Atrogin1), and *Trim63* (encoding MuRF1), which are *bona fide* atrogenes^48^, ^49^, SAA1 led to an increase in *Fbxo32* expression (*P* < 0.05), whereas the expression of *Fbxo30* and *Trim63* remained unchanged in differentiated C2C12 myotubes (*Figure* 1A).

To investigate if polymicrobial sepsis affects the expression of SAA receptors, we studied their expression in tibialis anterior and gastrocnemius/plantaris of mice subjected to CLP surgery. Muscle of sham‐operated mice was used as control. We found an increased expression of *Tlr2*, *Tlr4*, *Cd36*, and *Vimp* in both muscles of CLP compared with sham‐operated mice. In contrast, *P2rx7* was reduced in gastrocnemius/plantaris but remained unchanged in tibialis anterior of septic mice. Sepsis had no effect on *Scarb1* expression in both muscles investigated (*Figure* 1B). In order to investigate if the atrophy program is activated in skeletal muscle during sepsis, we quantitated the expression of *Fbxo30*, *Fbxo32*, and *Trim63* in both muscles of sham and CLP mice. *Fbxo30*, *Fbxo32*, and *Trim63* expression was increased in both muscles of septic mice, compared with sham controls, indicating that sepsis leads to an activation of the atrophy program in muscle (*Figure* 1B). Earlier, we had shown that ICUAW patients showed elevated amounts of SAA1 in skeletal muscle compared with controls.^11^ Therefore, we investigated whether or not *TLR2* and *TLR4* are also upregulated in vastus lateralis of ICUAW patients and used biopsy specimens from vastus lateralis of patients that underwent elective orthopaedic surgery as a control.^11^ QRT‐PCR showed a significant upregulation of *TLR2* and *TLR4* in muscle of ICUAW patients compared with controls (*Figure* 1C). In summary, our data show that all SAA receptors investigated are expressed in cultivated myocytes and in mouse skeletal muscle. The expression of *Tlr2*, *Tlr4*, *Cd36*, and *Vimp* increased in skeletal muscle of septic mice. Because SAA1 induced the expression of *Tlr2* in cultivated muscle cells and we found an increased expression of *Tlr2* and *Tlr4* in muscle of septic mice and critically ill patients, we focused on these SAA1 receptors for further analyses.

**Figure 2:**
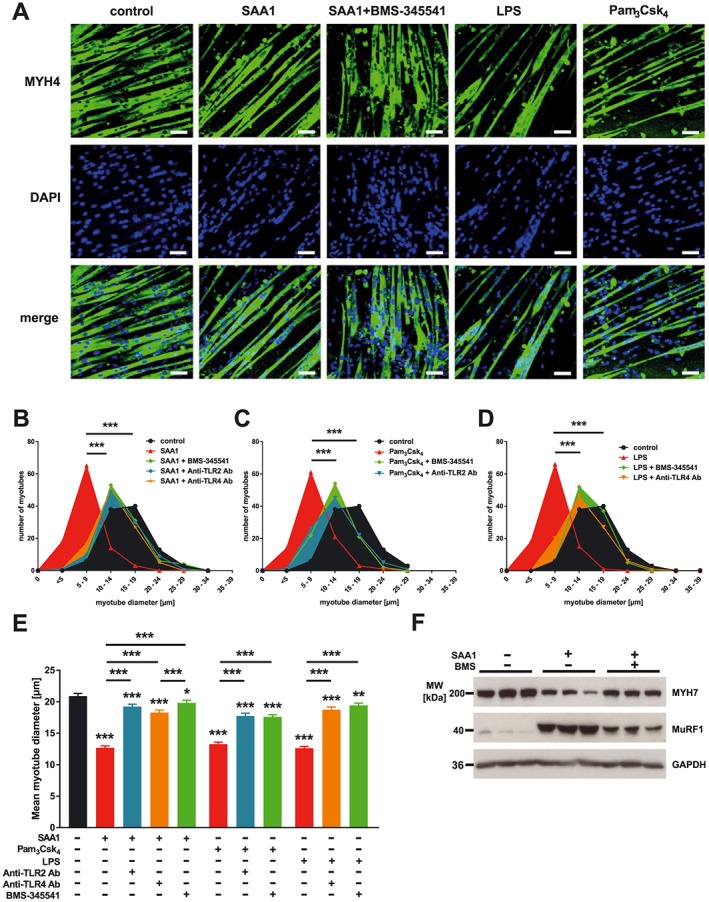
SAA1 causes atrophy of C2C12 myotubes via Toll‐like receptor (TLR) 2 and 4, in an NF‐κB dependent manner. C2C12 cells were differentiated for 5 days and treated with SAA1 (10 μg/mL), Pam_3_Csk_4_ (50 ng/mL), lipopolysaccharide (LPS; 1 μg/mL), anti‐TLR2 antibody (10 μg/mL), anti‐TLR4 antibody (20 μg/mL), or BMS‐345541 (5 μM), as indicated, for 72 h. (A) Immunohistochemistry with anti‐myosin heavy chain 4 (MyHC4, clone MF20) antibody. Nuclei were stained with DAPI. Scale bar = 100 μm. (B–D) Frequency distribution histograms of myotube diameters are shown (*n* = 100 cells per condition). (E) Mean myotube diameter. Data are presented as mean ± SEM. *P ≤ 0.05, **P ≤ 0.01, and ***P ≤ 0.001. (F) Western blot analysis with anti‐myosin heavy chain 7 (MyHC7; clone NOQ7.5.4D) and anti‐MuRF1antibody. Specificity of the anti‐MuRF1 antibody was tested by western blot analysis using tissue lysates of tibialis anterior muscle from wild type, *Trim63*/MuRF1 mutant and *Trim54*/MuRF3//*Trim63*/MuRF1 double mutant mice and of lysates from C2C12 cells following siRNA mediated knockdown of MuRF1 (data not shown). For more information, please refer to Nowak *et al*.^47^ GAPDH was used as loading control.

To test the hypothesis that SAA1 mediates its atrophic effects through TLR2 and TLR4 on myocytes, we treated C2C12 myotubes with SAA1, performed immunocytochemistry with anti‐myosin antibody, and measured myotube diameters as indicator for atrophy. We found that SAA1‐treatment caused myotube atrophy (*Figure* 2A) as indicated by a leftward‐shift of the frequency‐distribution histogram (*Figure* 2B) and a decrease in the mean myotube diameter (*Figure* 2E). Recently, we reported that inflammation causes a decrease in myosin heavy chain (MyHC) proteins in muscle and that the ubiquitin proteasome system, especially the muscle specific E3‐ligase MuRF1, was involved.^12^, ^34^ We therefore next investigated whether SAA1‐induced atrophy coincided with a decrease in MyHC and an increase in MuRF1 protein content. Western blot analysis showed that SAA1‐treatment led to a decrease in Myh7 and an increase in MuRF1 protein contents in C2C12 myotubes (*Figure* 2F). To test if SAA1‐induced atrophy is mediated by TLR2 and TLR4, we exposed C2C12 myotubes to SAA1 in the presence or absence of anti‐TLR2 and anti‐TLR4 antibody, respectively. Immunocytochemistry and measurements of myotube diameters showed that anti‐TLR2 and anti‐TLR4 antibody attenuated SAA1‐induced atrophy (*Figure* 2B and 2E). To investigate if both TLR‐receptors individually are capable to cause myocyte atrophy, we exposed C2C12 myotubes to the TLR2‐agonist Pam_3_Csk_4_ and the TLR4‐agonist lipopolysaccharide (LPS), respectively, and tested their atrophic response after addition of either anti‐TLR2 or anti‐TLR4 antibody. Both Pam_3_Csk_4_ and LPS caused myotube atrophy, which was blocked by their respective inhibitors. These data indicate that TLR2 and TLR4 are equally effective to induce myotube atrophy (*Figure* 2A and 2C–2E).

**Figure 3:**
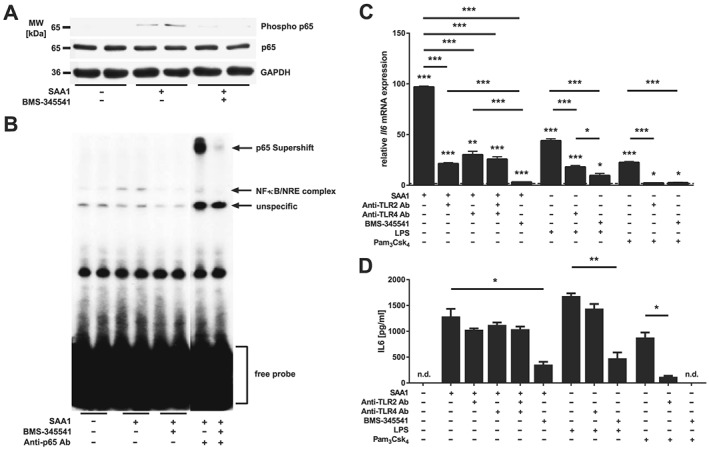
SAA1 increases *Il6* expression and secretion via Toll‐like receptor (TLR) 2, TLR4 and NF‐κB p65. (A) C2C12 cells were treated with SAA1 (10 μg/mL) for 25 min in the presence or absence of BMS‐345541 (25 μM). Western blot analysis with anti‐phospho p65 antibody and anti‐p65 antibody are shown. GAPDH was used as loading control. (B) C2C12 cells were treated with SAA1 (10 μg/mL) for 25 min in the presence or absence of BMS‐345541 (25 μM). NF‐кB p65/NF‐κB‐response element complex formation was analysed by EMSA. (C) Five‐day‐differentiated C2C12 myotubes were preincubated with anti‐TLR2 antibody (10 μg/mL), anti‐TLR4 antibody (20 μg/mL), or BMS‐345541 (25 μM) as indicated. Afterwards cells were treated with SAA1 (10 μg/mL), LPS (1 μg/mL), or Pam_3_Csk_4_ (50 ng/mL) as indicated. qRT‐PCR analysis of *Il6*. mRNA expression was normalized to *Gapdh*. Control values were set to 1 and are indicated as dotted line. Data are presented as mean fold change ± SEM. (D) Quantification of IL6 in cell culture supernatants by ELISA. Cells were preincubated with anti‐TLR2 antibody (10 μg/mL), anti‐TLR4 antibody (20 μg/mL), or BMS‐345541 (25 μM) as indicated prior to SAA1 treatment (10 μg/mL). Data are presented as the mean ± SEM. n.d., not detectable; *P ≤ 0.05, **P ≤ 0.01, and ***P ≤ 0.001.

Because TLR2 and TLR4 activate NF‐κB signalling in immune cells,^50^, ^51^ we investigated if SAA1 activates NF‐κB via TLR2 and TLR4 in myocytes. Western blot analysis showed that SAA1‐treatment caused an increased phosphorylation of the NF‐κB transcription factor p65 in C2C12 myotubes (*Figure* 3A). Electrophoretic mobility shift assay (EMSA) revealed that SAA1‐treatment led to an increased binding of activated NF‐κB p65 to NF‐ĸB response elements (NRE) (*Figure* 3B). Addition of an anti‐p65 antibody to the EMSA‐assay resulted in a super‐shift of the NF‐κB p65/NRE complex underscoring the specificity of this assay (*Figure* 3B). To further investigate the NF‐κB p65 pathway, we used the IκB kinase (IKK) inhibitor BMS‐345541 that abolishes IKK‐dependent phosphorylation of p65 and of IκBα and subsequent degradation of IκBα and activation of NF‐κB.^41^ We found that SAA1‐induced phosphorylation of NF‐κB p65 was attenuated by BMS‐345541 (*Figure* 3A) indicating that IKK is involved in this process. Likewise, BMS‐345541 abolished SAA1‐induced NF‐κB p65/NRE complex formation and the super‐shift in EMSA (*Figure* 3B).

Although NF‐κB p65 mediates denervation and tumour‐induced atrophy^52^, ^53^ and its inhibition prevents muscle atrophy in mice with acute lung injury,^54^ whether or not NF‐κB p65 mediates SAA1‐induced atrophy is unknown. We therefore treated C2C12 myotubes with SAA1 in the presence or absence of BMS‐345541 and quantitated the atrophic response. Indeed, BMS‐345541 abolished SAA1‐induced atrophy (*Figure* 2A, 2B, and 2E), which was accompanied by an increase in Myh7 and a decrease in MuRF1 proteins as shown by western blot (*Figure* 2F). These data indicate that SAA1‐induced atrophy is mediated by TLR2 and TLR4 and that the NF‐κB p65 pathway is involved.

Recently, we reported that ICUAW patients and septic mice show increased muscular IL6 and SAA1 levels.^11^ Interestingly, IL6‐treatment causes an increase in SAA1 expression in myocytes,^11^ and conversely SAA1 induces IL6 in fibroblasts.^33^ These data suggested a positive feedback loop between SAA1 and IL6. To test if this feedback loop is present in myocytes and depends on TLR2‐mediated and TLR4‐mediated NF‐κB activation, we investigated if SAA1 induces IL6 in these cells. Using qRT‐PCR, we found that SAA1 increased *Il6* expression in C2C12 myotubes. Anti‐TLR2 and TLR4 antibodies, as well as BMS‐345541 treatment, attenuated SAA1‐induced *Il6* expression (*Figure* 3C). ELISA‐based quantification of the IL6 content in C2C12 cell culture supernatant revealed that SAA1‐induced IL6‐secretion was diminished by BMS‐345541 but remained unaffected by anti‐TLR2 and TLR4 antibody (*Figure* 3D). To further test if TLR2 and TLR4 activation regulate both IL6 expression and secretion, we treated C2C12 myotubes with the TLR2‐agonist Pam_3_Csk_4_ and the TLR4‐agonist lipopolysaccharide (LPS), respectively, in the presence or absence of anti‐TLR2 or TLR4 antibody. The anti‐TLR2 antibody reduced Pam_3_Csk_4_‐induced IL6 expression and secretion. In contrast, the anti‐TLR4 antibody did not inhibit LPS‐induced IL6‐secretion (*Figure* 3C and 3D). BMS‐345541 attenuated Pam_3_Csk_4_‐induced and LPS‐induced *Il6* expression and secretion (*Figure* 3C and 3D). These data suggest that SAA1‐induced *Il6* expression is mediated by TLR2 and TLR4 and that the NF‐κB p65 pathway is involved in this process.

### Sepsis‐induced muscle atrophy is reduced by BMS‐345541 treatment *in vivo*

**Figure 4:**
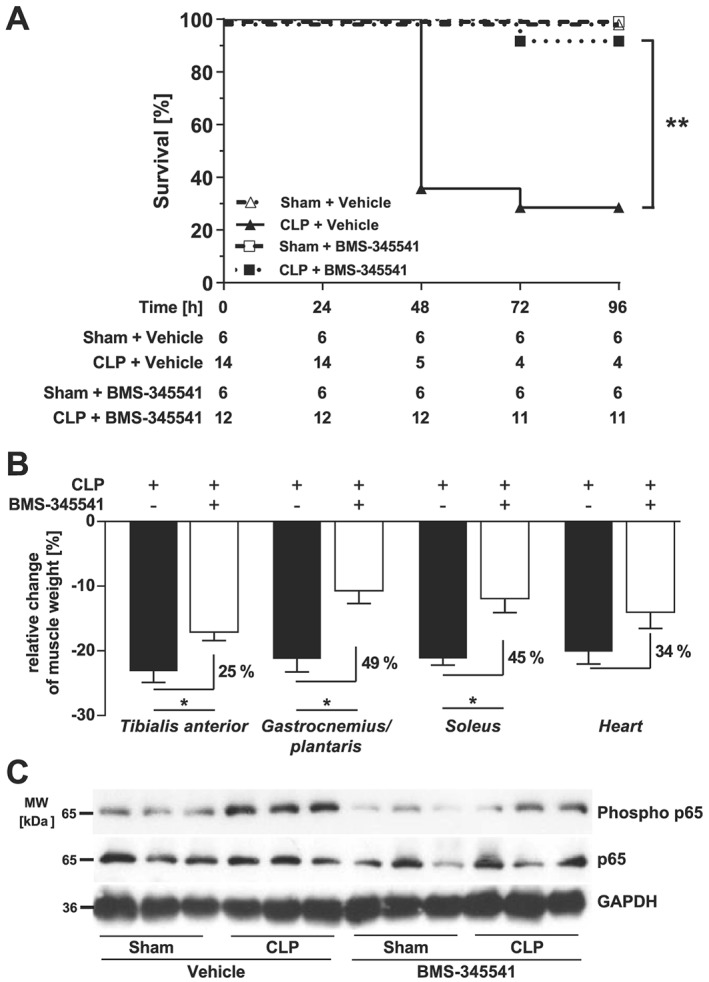
BMS‐345541 treatment increases survival and attenuates muscle atrophy in septic mice *in vivo*. (A–C) Eighteen‐week‐old male WT mice were either sham or CLP operated as indicated. Sham‐operated and CLP‐operated mice were divided into two groups each which then received either vehicle (5% TWEEN 20 in distilled H_2_O, pH 7.4) or BMS‐345541 30 min prior to (30 mg/kg body weight) and 6 h after (15 mg/kg body weight) surgery p.o.; experimental groups are as follows: sham + vehicle (*n* = 6), CLP + vehicle (*n* = 14), sham + BMS‐345541 (*n* = 6), and CLP + BMS‐345531 (*n* = 12). After 96 h, all mice were sacrificed. (A) Survival of all animals. Statistical analysis was performed using the log‐rank test. **P ≤ 0.01. (B) Weights of tibialis anterior, gastrocnemius/plantaris, soleus, and the heart normalized to tibia length. Data are presented as mean ± SEM. *P ≤ 0.05. (C) Western blot analysis of tibialis anterior with anti‐phospho‐p65 and anti‐p65 antibody. GAPDH was used as loading control.

Because our data indicated that SAA1 causes myocyte atrophy via the TLR2/TLR4/NF‐κB pathway, we hypothesized that inhibition of the NF‐κB pathway could prevent muscle atrophy in septic mice. To test this hypothesis, we subjected male WT mice to sham or CLP surgery and treated them with either vehicle or BMS‐345541, which were administered by oral gavage.^41^ BMS‐345541 treatment resulted in highly significant improvement of survival of CLP‐operated compared with vehicle‐treated septic mice (*Figure* 4A). Sepsis‐induced reduction in tibialis anterior (−25%, *P* < 0.05), gastrocnemius/plantaris (−49%, *P* < 0.05), and *soleus* (−45%, *P* < 0.05) weights was inhibited by BMS‐345541. Sepsis caused a reduction in heart weight as well (sham + vehicle vs. sham + BMS‐345541: −20.4%, *P* < 0.05; sham + BMS‐345541 vs. CLP + BMS‐345541: −14.4%, *P* < 0.05). BMS‐345541 treatment was accompanied by a reduced cardiac weight loss which was not significant (*P* = 0.3) (*Figures* 4B and S1). As expected, western blot analysis showed an increased NF‐κB p65 phosphorylation in tibialis anterior of septic compared with sham operated mice, which was attenuated by BMS‐34551 (*Figure* 4C). Our data show that inhibition of sepsis‐induced NF‐κB activation by BMS‐345541 improves survival and attenuates muscular weight loss during sepsis.

**Figure 5:**
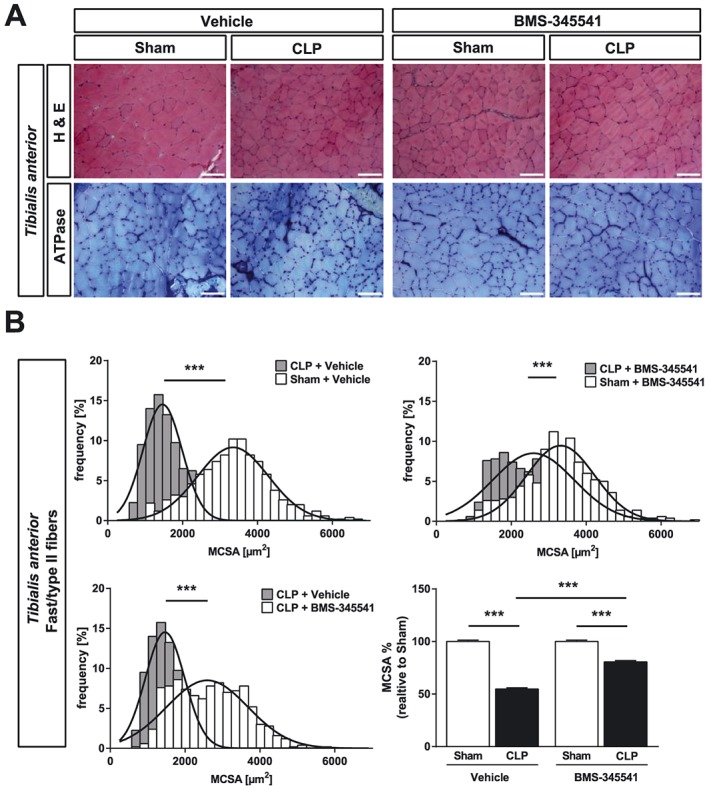
BMS‐345541 treated mice are protected from sepsis‐induced atrophy of the tibialis anterior muscle. Eighteen‐week‐old male WT mice were either sham or CLP operated as indicated. Per surgical group mice were divided into two groups each which than received either vehicle (5% TWEEN 20 in distilled H_2_O, pH 7.4) or BMS‐345541 30 min prior to (30 mg/kg body weight) and 6 h after (15 mg/kg body weight) surgery p.o.; experimental groups are as follows: sham + vehicle (*n* = 6), CLP + vehicle (*n* = 4), sham + BMS‐345541 (*n* = 6), and CLP + BMS‐345531 (*n* = 11). After 96 h, the mice were sacrificed. (A) Haematoxylin and eosin (H&E) and ATPase staining of histological cross sections from tibialis anterior (TA). Scale bar = 100 μm. (B) Quantification of fast/type II myofiber cross‐sectional area (MCSA) from metachromatic ATPase staining (as depicted in panel A). One hundred myofibers per animal were measured. ***P ≤ 0.001.

**Figure 6:**
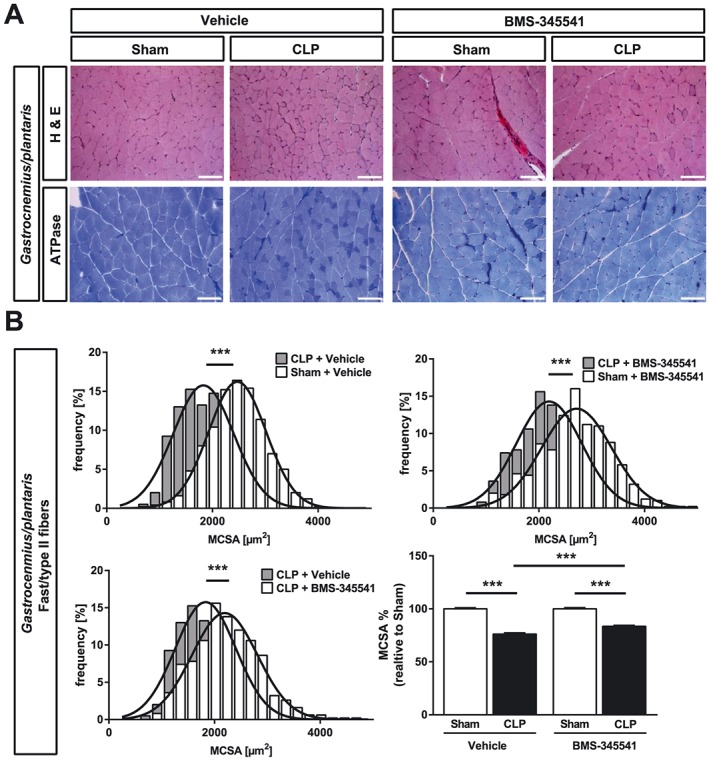
BMS‐345541 treated mice are protected from sepsis‐induced muscle atrophy of the gastrocnemius/plantaris. Eighteen‐week‐old male WT mice were either sham or CLP operated as indicated. Per surgical group, mice were divided into two groups each which then received either vehicle (5% TWEEN 20 in distilled H_2_O, pH 7.4) or BMS‐345541 30 min prior to (30 mg/kg body weight) and 6 h after (15 mg/kg body weight) surgery p.o.; experimental groups are as follows: sham + vehicle (*n* = 6), CLP + vehicle (*n* = 4), sham + BMS‐345541 (*n* = 6), and CLP + BMS‐345531 (*n* = 11). After 96 h, the mice were sacrificed. (A) Haematoxylin and eosin (H&E) and ATPase stain of histological cross sections from gastrocnemius/plantaris (GP). Scale bar = 100 μm. (B) Quantification of fast/type II myofiber cross‐sectional area (MCSA) from metachromatic ATPase staining (as depicted in panel A). One hundred myofibers per animal were measured. ***P ≤ 0.001.

**Figure 7:**
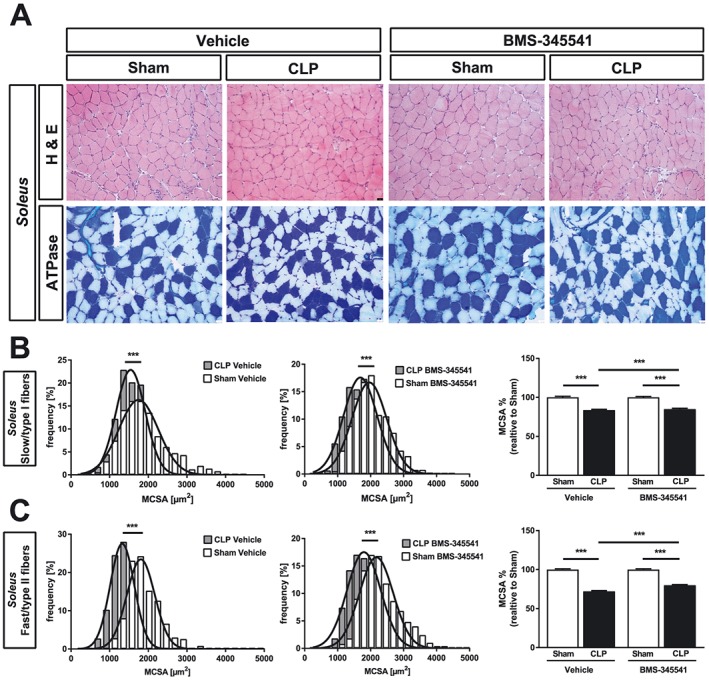
BMS‐345541 treated mice are protected from sepsis‐induced muscle atrophy of the *soleus*. Eighteen‐week‐old male WT mice were either sham or CLP operated as indicated. Per surgical group, mice were divided into two groups each which then received either vehicle (5% TWEEN 20 in distilled H_2_O, pH 7.4) or BMS‐345541 30 min prior to (30 mg/kg body weight) and 6 h after (15 mg/kg body weight) surgery p.o.; experimental groups are as follows: sham + vehicle (*n* = 6), CLP + vehicle (*n* = 4), sham + BMS‐345541 (*n* = 6), and CLP + BMS‐345531 (*n* = 11). After 96 h, the mice were sacrificed. (A) Haematoxylin and eosin (H&E) and ATPase stain of histological cross sections from *soleus*. Scale bar = 100 μm. (B) Quantification of slow/type I and fast/type II myofiber cross‐sectional area (MCSA) from metachromatic ATPase staining (as depicted in panel A). One hundred myofibers per animal were measured. ***P ≤ 0.001.

Recently, others and we showed that primarily fast‐twitch fibers undergo atrophy in ICUAW patients and septic mice.^12^, ^34^ Therefore, we tested if BMS‐345541 protects fast/type‐II myofibers from atrophy in sepsis. Analyses of tibialis anterior and gastrocnemius/plantaris cross sections and quantification of myocyte cross‐sectional area (MCSA) showed that CLP treated animals had a pronounced atrophy of fast/type‐II myofibers (*Figures* 5A, 5B, 6A, and 6B). CLP operated mice that were treated with BMS‐345541 showed less myofiber atrophy compared with vehicle‐treated CLP mice as indicated by a significantly lower reduction in MCSA in tibialis anterior and gastrocnemius/plantaris (*Figures* 5A, 5B, 6A, and 6B). Because the soleus contains both slow/type‐I and fast/type‐II myofibers, we performed histological cross sections of the soleus and quantitated the MCSA for both fiber types following metachromatic ATPase staining. We found a reduction in the MCSA of slow/type‐I (sham + vehicle vs. CLP + vehicle: −17%, *P* < 0.001; sham + BMS‐345541 vs. CLP + BMS‐345541: −15%, *P* < 0.001) and fast/type‐II myofibers (sham + vehicle vs. CLP + vehicle: −28%, *P* < 0.001; sham + BMS‐345541 vs. CLP + BMS‐345541: −20%, *P* < 0.001). We found a significantly lower MCSA reduction of slow/type‐I (*P* < 0.001) and fast/type‐II myofibers (*P* < 0.001) in *soleus* of BMS‐345541‐treated septic mice (*Figure* 7A and 7B). Our results show that fast/type‐II myofibers are more susceptible than slow/type‐I myofibers to sepsis‐induced atrophy and that BMS‐345541 treatment protects from sepsis‐induced myofiber atrophy in mice *in vivo*.

**Figure 8:**
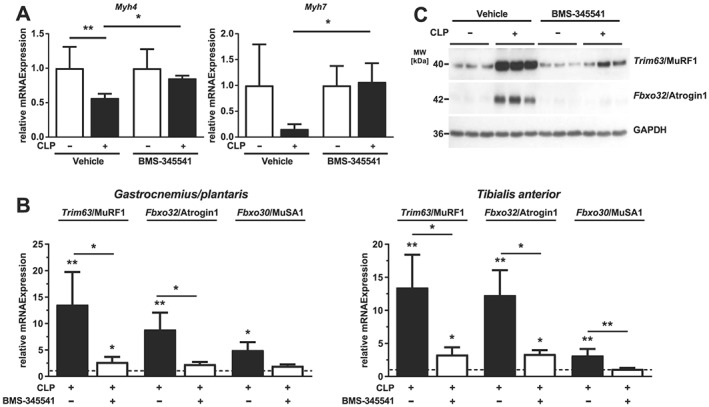
BMS‐345541 treatment attenuates sepsis‐induced protein degradation. Eighteen‐week‐old male WT mice were Sham or CLP operated. Mice received vehicle or BMS‐345541 (30 mg/kg) 30 min prior surgery and 6 h later vehicle or BMS‐345541 (15 mg/kg) by oral gavage. After 96 h, the mice were sacrificed. (A and B) Sham + vehicle (*n* = 6), CLP + vehicle (*n* = 4), sham + BMS‐345541 (*n* = 6), and CLP + BMS‐345531 (*n* = 11). (A) qRT‐PCR analysis of myosin heavy chain (*Myh*) *4* and *Myh7* expression in tibialis anterior muscle. mRNA expression was normalized to *Gapdh*. (B) qRT‐PCR analysis of *Trim63*/MuRF1, *Fbxo32*/Atrogin1, and *Fbxo30*/MuSA1 expression in tibialis anterior and gastrocnemius/plantaris muscle. mRNA expression was normalized to *Gapdh*. Data are presented as fold change ± SEM. *P ≤ 0.05 and **P ≤ 0.01. (C) Western blot analysis of proteins isolated from tibialis anterior with anti‐MuRF1 and anti‐Atrogin1 antibody. GAPDH was used as loading control.

We had also observed that an increased protein degradation and a decreased protein synthesis contributes to muscle atrophy in ICUAW patients and septic mice.^12^ To test if the inhibition of septic muscle atrophy by BMS‐345541 was accompanied by an improved protein homeostasis, we quantitated *Myh4* and *Myh7* expression in tibialis anterior by qRT‐PCR. Sepsis led to a decreased *Myh4* expression (sham + vehicle vs. CLP + vehicle: −56%, *P* < 0.01; sham + BMS‐345541 vs. CLP + BMS‐345541: −15%, n.s.), which was attenuated by 34% by BMS‐345541 (*P* < 0.05). *Myh7* expression followed the same trend that did not reach significance (*Figure* 8A). To investigate the effect of BMS‐345541 treatment on protein degradation, we quantified the expression of *Fbxo30*, *Fbxo32*, and *Trim63* in tibialis anterior and gastrocnemius/plantaris. CLP significantly increased *Fbxo32* and *Trim63* expression, and BMS‐345541 treatment attenuated this response in both muscles. *Fbxo30* was also significantly increased in tibialis anterior and gastrocnemius/plantaris of vehicle‐treated septic mice but remained unchanged in BMS‐345541 treated animals (*Figure* 8B). The Atrogin1/*Fbxo32* and MuRF1/*Trim63* protein content increased during sepsis in tibialis anterior and BMS‐345541 attenuated this response (*Figure* 8C). Our data indicate that the attenuation of sepsis‐induced atrophy by BMS‐345541 involved an improved protein homeostasis.

Increased NF‐κB activity plays a pivotal role for the regulation of immune cell function during a bacterial insult.^55^, ^56^ Indeed, immune cell activation is important for bacterial clearance in sepsis.^42^, ^57^ To investigate if BMS‐345541 treatment affected immune cells, we performed flow cytometric analyses of splenocytes. Sepsis caused an increase in spleen weight, which was unaffected by BMS‐345541 (*Figure* S2). The total numbers and proportions of dendritic cells (CD11c^hi^MHCII^+^), B‐cells (CD19^+^B220^+^), and natural killer cells (NK, NK1.1^+^) decreased (*Figure* S3A–S3C), while neutrophil numbers (Ly6G^+^) increased in septic mice (*Figure* S3D). We also observed a significant reduction in the proportions of CD3^+^ T‐cells, which was due to decreased percentages of CD4^+^ helper T‐cells in the spleens of septic mice, whereas CD8^+^ cytotoxic T‐cells remained unchanged (*Figure* S4A–S4C). Finally, percentages of Foxp3^+^CD25^+^CD4^+^ regulatory T‐cells were increased, and Foxp3^‐^/CD4^+^ effective T‐cells were decreased in the spleen of septic mice, and both T‐cell subpopulations were activated, as shown by the increased CD69 expression. In the case of vehicle‐treated animals, this increase did not reach significance (*Figure* S5A–S5C). Notably, BMS‐345541 treatment had no significant effect on sepsis‐induced alterations of numbers, proportions, and activation of splenic immune cells, indicating that its strong impact on survival and muscle atrophy were independent of the immune cell responses to sepsis.

## Discussion

Serum amyloid A1 (SAA1) accumulation in muscle is associated with ICUAW.^11^ However, neither the putative receptors for SAA1 nor the involved pathways have been described in myocytes or muscle. We identified Toll‐like receptors 2 and 4 as SAA1 receptors on myocytes *in vitro*. We show that SAA1 via TLR2 and TLR4 activates the NF‐κB p65 pathway that mediates the SAA1‐response of myocytes. Specifically, we demonstrate that SAA1‐induced myocyte atrophy is attenuated by blocking either TLR2 or TLR4 or by inhibition of the NF‐κB p65 pathway with BMS‐345541 *in vitro*. We show that inhibition of NF‐κB activity by BMS‐345541 increased survival and reduced muscle atrophy in a mouse model of polymicrobial sepsis *in vivo*. Our findings suggest that inhibition of NF‐κB during sepsis may increase survival and attenuate SAA1‐induced muscle atrophy.

Although the function of SAA1 is well described for liver and immune cells,^58^ any role in muscle is less well understood. For example, SAA1 can bind to several receptors, such as TLR2, TLR4, CD36, P2RX7, VIMP, and SCARB1, which are expressed on hepatocytes, keratinocytes, fibroblasts, and macrophages.^25^, ^26^, ^27^, ^28^, ^30^, ^31^, ^33^, ^59^ Whether or not these receptors are involved in SAA1 signalling in myocytes was uncertain. Here, we show that TLR2, TLR4, CD36, P2RX7, VIMP, and SCARB1 are also expressed in myocytes and muscle and that muscular expression of *TLR2*, *TLR4*, *CD36*, and *VIMP* increases during sepsis. We found that SAA1 causes myocyte atrophy *in vitro*, which was mediated by TLR2 and TLR4 as well as NF‐κB. Together with our recent report showing that SAA1 expression, synthesis, and secretion increase in muscle of ICUAW patients and septic mice, our data indicate that muscle is targeted by SAA1 in a paracrine manner and that TLR2, TLR4, and NF‐κB are involved in SAA1‐induced atrophy.

TLR2 was shown to mediate SAA1‐induced *Il6* expression in fibroblasts^33^ and to mediate *Il6* expression and atrophy in myocytes.^8^ Activation of TLR2 and TLR4 by specific agonists were found to mediate IL6 secretion from C2C12 myotubes.^60^ Recently, we reported that SAA1 increases *Il6* expression in murine and human myocytes *in vitro*.^11^ In addition, IL6 expression is increased in muscle of ICUAW patients and septic mice.^11^, ^12^ We found that muscular TLR2 and TLR4 expression is increased during sepsis in patients and mice, that SAA1 increases *Tlr2* and *Tlr4* expression in myotubes, and that SAA1 via TLR2 and TLR4 increases *Il6* expression in myocytes. Based on these data, we hypothesize that during sepsis, SAA1 participates in a positive feedback loop of self‐perpetuating inflammation in muscle not only by increasing IL6 expression but also by increasing the expression of its own receptors, which further augments IL6 expression. Moreover, we showed that SAA1 induces IL6 expression in myocytes and *vice versa*, which further supports a self‐augmenting positive feedback loop in muscle during inflammation. Considering that SAA1 accumulation correlates with muscle atrophy in ICUAW patients, these feedback mechanisms could worsen IL6‐mediated atrophy.

Because LPS elevates NF‐κB‐dependent gene expression via TLR4,^61^, ^62^ we investigated whether SAA1 activates this pathway in myocytes. We show that SAA1 activates the canonical NF‐κB p65 signalling pathway via TLR2 and TLR4 resulting in an increased expression of NF‐κB p65 target genes and myocyte atrophy. For our analyses, we used the highly selective IKK inhibitor BMS‐345541. IKK phosphorylates IκBα and NF‐κB p65, leading to the proteasomal degradation of IκBα and release of active NF‐κB.^63^ Indeed, BMS‐345541 decreased SAA1‐induced NF‐κB p65 phosphorylation, inhibited formation of NF‐κB‐DNA complexes, and attenuated SAA1‐induced *Il6* expression in myotubes as well as NF‐κB p65 phosphorylation in muscle of septic mice. These data indicate that BMS‐345541 effectively inhibited NF‐κB activity in myocytes. In addition, BMS‐345541 attenuated myotube atrophy as well as the SAA1‐induced molecular changes consistently described as atrophic response with an increase in MuRF1 and a decrease in MyHC.^34^, ^48^, ^64^, ^65^, ^66^ Our results indicate that SAA1‐induced atrophy was mediated by NF‐κB. Of note, NF‐κB p65 was shown to mediate denervation and tumour‐induced muscle atrophy,^52^, ^53^ and its inhibition prevented atrophy in a mouse model of acute lung injury.^54^ Our data corroborate and extend these findings (i) by demonstrating that NF‐κB has an important role in sepsis‐induced atrophy as well and (ii) by identifying SAA1 as a facilitator of the NF‐κB p65 pathway, which mediates most of its effects on myocytes *in vitro*.

To investigate if NF‐κB inhibition prevents sepsis‐induced muscle atrophy *in vivo*, we tested BMS‐345541^41^ in the CLP mouse model. The expression of many pro‐inflammatory genes is regulated by NF‐κB. NF‐κB normally resides in the cytoplasm of unstimulated cells as an inactive complex with a member of the IκB inhibitory protein family. This class of protein includes IκBα, IκBβ, and IκBε, which all form a complex with NF‐κB (for a review, see Whiteside and Israel^67^). Stimulation of cells with compounds that activate NF‐κB‐dependent gene expression results in the phosphorylation of IκBα at Ser‐32 and Ser‐36.^68^ This is critical for subsequent ubiquitination and proteolysis of IκBα, which then leaves NF‐κB free to translocate to the nucleus and promote gene expression.^69^ Analogous serines have been identified in both IκBβ and IκBε, and phosphorylation at these residues appears to regulate the proteolytic degradation of these proteins by a mechanism similar to that of IκBα.^70^ Phosphorylation of IκBα at Ser‐32 and Ser‐36 is mediated by a high molecular mass (500–900 kDa) multisubunit IκB kinase (termed IKK).^71^ Two catalytic subunits (termed IKK‐1 and IKK‐2) of IKK have recently been identified.^72^ Several working groups have shown that all known pro‐inflammatory stimuli, including cytokines, viruses, and LPS, require IKK for NF‐κB activation (for a review, see Ghosh and Karin^73^). Previously, BMS‐345541, a highly selective cell permeable IKK‐2 and IKK‐1 inhibitor that inhibits NF‐κB‐dependent transcription of pro‐inflammatory cytokines both *in vitro* and *in vivo* was identified. Subsequently, BMS‐345541 was shown to attenuate LPS‐induced production of TNF, IL1β, IL8, and IL6 in THP‐1 cells.^41^ BMS‐345541 was also shown to abolish TNF‐stimulated IκBα‐phosphorylation in THP‐1 cells^41^ and the prostate cancer cell line PC‐3.^46^ BMS‐345541 was previously shown to reduce CLP and LPS‐induced lung injury,^42^, ^43^ to reduce inflammation in rats with spinal cord injury, and to suppress inflammation in a model of cardiac graft rejection.^44^, ^45^ Due to its high bioavailability^41^ and its anti‐inflammatory characteristics without impairing immune cell activation^42^ and bacterial clearance, BMS‐345541 was well suited for our studies. Indeed, we show that NF‐κB p65 was activated in muscle of septic mice, which is in accordance with previously published work demonstrating NF‐κB activation in muscle of CLP operated rats.^74^ Next, we confirmed that BMS‐345541 prevented activation of NF‐κB p65 in muscle of septic mice, which is in line with a recent report where BMS‐345541 was shown to inhibit LPS‐induced NF‐κB‐dependent gene expression in mice^41^ and proves its biological activity. Interestingly, BMS‐345541 treatment resulted in a 63% percent higher survival rate of septic mice. Because increased NF‐κB activity in peripheral blood mononuclear cells predicted fatal outcome in septic patients and inhibition of the IKK‐NF‐κB axis was shown to increase survival in an LPS‐induced toxic shock model in mice,^75^ this result was somewhat expected. Importantly, BMS‐345541 reduced the degree of sepsis‐induced muscle atrophy. Accordingly, sepsis‐induced reduction in *Myh4* expression and induction of *Trim63*/MuRF1 and *Fbxo32*/Atrogin1 expression was reversed by BMS‐345541. MuRF1 mediates proteasomal degradation of muscular proteins, like MyHC2 and 4,^64^ and as an atrophy marker is increased in muscle of septic patients and mice.^34^, ^37^ A previous report showed that mice overexpressing activated IKK2 in muscle, which increases NF‐κB activity, displayed increased MuRF1 expression and aggravated muscle atrophy. When these mice were crossed to MuRF1 knockout mice, atrophy was greatly reduced.^52^ Together with our report, these data suggest that NF‐κB signalling contributes to sepsis‐induced muscle atrophy, which involves MuRF1‐dependent protein degradation.

Our data implicate that inhibition of NF‐κB by BMS‐345541 could be effective to decrease muscle wasting in sepsis. Because NF‐κB is a key regulator of inflammatory responses and involved in other forms of muscle wasting,^52^, ^53^, ^76^ we hypothesize that BMS‐345541 could be useful to prevent or treat other forms of muscle wasting as well. For example, end‐stage cancer patients often suffer from cachexia, which is accompanied with a poor prognosis. Among the best studied inflammatory cytokines promoting cancer cachexia are TNF, IL6,^77^ IL1, and interferon gamma,^78^ which are all elevated^79^ and may together trigger muscle wasting by increasing NF‐κB activity.^80^, ^81^, ^82^ For instance, TNF promotes anorexia^83^ and skeletal muscle wasting mainly through activation of the NF‐κB pathway.^84^ However, the precise mechanism how NF‐κB mediates muscle wasting in cancer is less well understood. One explanation is that NF‐κB signalling and UPS‐mediated protein degradation are closely connected to each other. On one side, NF‐κB increases the expression of atrogenes and proteasome‐mediated protein degradation in muscle. On the other side, proteasome inhibitors interfere with the NF‐κB pathway because they inhibit IκB degradation, which in turn prevents NF‐κB activation.^85^ Accordingly, an increased *Fbxo32*/Atrogin1 gene expression^86^ and protein ubiquitination^86^ was associated with an activated UPS in a rat model of cachexia, induced by the Yoshida ascites hepatoma cells. In tumour‐bearing mice, this UPS activation was mediated by an increased NF‐κB activity, which stimulated *Trim63*/MuRF1 expression and muscle wasting.^52^ These data implicate that NF‐κB inhibition could be useful to prevent cancer cachexia. Indeed, synthetic double‐stranded oligodeoxynucleotides, which block NF‐κB binding to promoter regions, inhibited cachexia in a mouse tumour model.^87^ However, if BMS‐3445541 is useful to prevent muscle wasting in cancer needs to be shown in the future. Furthermore, because NF‐κB activation is important for denervation‐induced muscle wasting, many approaches were undertaken to reduce its activity to attenuate this phenotype. For example, misexpression of IκB that was used to inhibit NF‐κB activity reduced denervation‐induced muscle wasting in tumour‐bearing mice.^52^ Denervation‐induced atrophy was also attenuated in mice lacking IKKβ.^53^ Inhibition of NF‐κB by BMS‐3445541 could therefore be useful to prevent denervation‐induced muscle wasting as well.

Finally, and in agreement with previous reports,^42^ BMS‐345541 did not affect the immune cell responses to sepsis.^42^ In summary, our data indicate that inhibition of the IKK‐NF‐κB pathway holds promise to prevent both mortality and muscle atrophy in sepsis.

## Conclusions

**Figure 9:**
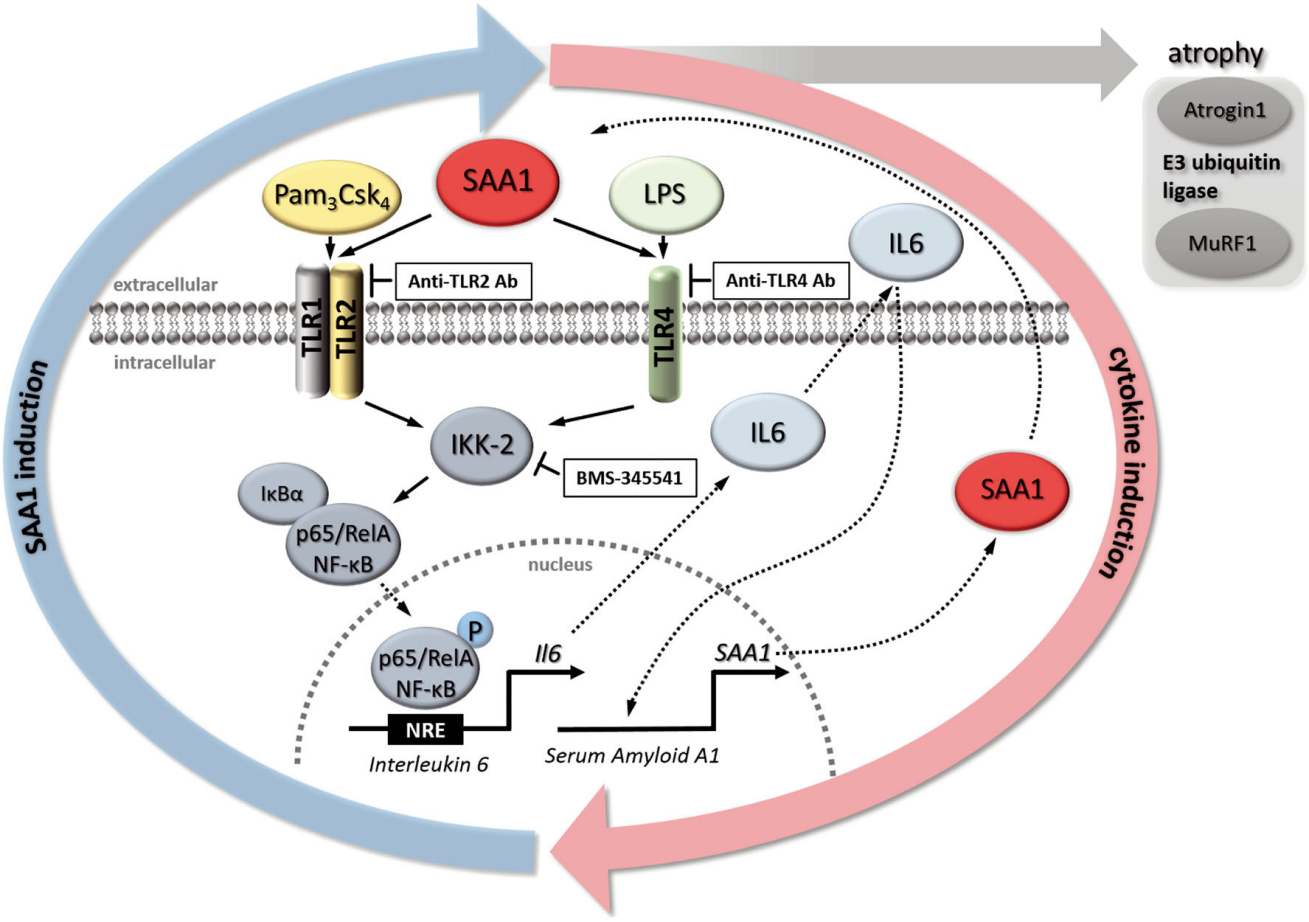
Proposed mechanism of serum amyloid A 1 (SAA1) induced muscle atrophy. SAA1 binds to muscular Toll‐like receptors (TLR) 2 and 4, which results in an activation of the canonical NF‐κB p65 pathway leading to NF‐κB p65/RelA phosphorylation and its translocation into the nucleus. In the nucleus, NF‐κB p65/RelA binds to NF‐κB response elements (NRE), which leads to increased expression and secretion of the pro‐inflammatory cytokine *Il6*. IL6 in turn induces expression and secretion of SAA1 resulting in a positive feedback loop. In addition, SAA1 causes an increase of MuRF1/*Trim63* and Atrogin1/*Fbxo32*, which mediate muscle atrophy in sepsis. Anti‐TLR2 or anti‐TLR4 antibodies inhibit SAA1‐induced *Il6* expression. The specific IκB kinase subunit IKK‐2 inhibitor BMS‐345541 inhibits SAA1‐induced and NF‐κB dependent gene expression, thereby reducing SAA1‐mediated atrophy and inhibiting the self‐augmenting SAA1‐IL6 feedback loop.

During sepsis, SAA1 is increased in muscle and directly acts on myocytes via the TLR2/TLR4/NF‐κB p65 axis. Blockade of SAA1‐mediated NF‐κB activation by BMS‐345541 attenuated atrophy and inhibited the SAA1‐IL6 feedback loop in myocytes *in vitro*. Likewise, BMS‐345541 increased survival and attenuated skeletal muscle atrophy in septic mice (*Figure* 9). We suggest that monitoring SAA1 and inhibition of the SAA1/TLR2/TLR4/NF‐κB atrophy pathway could ameliorate ICUAW. Further studies are needed to determine how the SAA1 pathway is mechanistically connected to other atrophy pathways such as MuRF1 and Atrogin1 that we found to be affected.

## Conflict of interest

A.H., M.K., C.P.‐T., M.T., M.W., S.S., K.S., I.J., M.N., E.J., T.S., B.M.B., C.S., S.W.‐C., C.B., and F.C.L. declare that they have no conflict of interest. J.F. has received research grants from the Deutsche Forschungsgemeinschaft. J.F. and S.B.F. have received research grants from the German Center for Cardiovascular Research.

## Authors' contributions

A.H., M.K., C.P.T., M.T., M.W., S.S., K.S., I.J., M.N., E.J. and T.S. designed, performed, and analysed experiments and discussed data. A.H., M.K., and J.F. prepared and edited the manuscript. B.B., S.B.F., C.S., S.W.‐C., C.B., F.C.L., and J.F. designed experiments, discussed data, and edited the manuscript. All authors declare that the submitted work has not been published before (neither in English nor in any other language) and that the work is not under consideration for publication elsewhere.

## Declarations

### Ethics approval and consent to participate

The institutional review board of the Charité approved the study, and written informed consent was obtained from legal proxy (ICU patients), or the patients themselves (Charité EA2/061/06; http://www.controlled-trials.com, ISRCTN77569430).

The *Landesamt für Gesundheit und Soziales*, Berlin, Germany (G207/13, G129/12), approved the animal studies (NIH publication No. 86‐23, revised 1985).

## Supporting information


**Data S1.** Supporting Information

**Figure S1.** Supporting Information

**Figure S2.** Supporting Information

**Figure S3.** Supporting Information

**Figure S4.** Supporting Information
