# Supplementary material for: Serum amyloid A1 mediates myotube atrophy via Toll‐like receptors
Source: J Cachexia Sarcopenia Muscle. 2019 Aug 23;11(1):103–19. doi: 10.1002/jcsm.12491 (PMC7015249; doi:10.1002/jcsm.12491)
Supplement: Supplementary file 1 — Data S1. Supporting Information [file JCSM-11-103-s002.docx]

**SUPPLEMENTARY METHODS**

**Patient samples.**

The institutional review board of the Charité Universitätsmedizin Berlin, Germany, approved the study, and written informed consent was obtained from legal proxy (ICU patients), or the patients themselves (Charité EA2/061/06; http://www.controlled-trials.com, ISRCTN77569430). Clinical data were reported previously [1]. Here we analysed the expression of Toll-like receptor 2 (*TLR2*), *TLR4* and Glyceraldehyde 3-phosphate dehydrogenase (*GAPDH*) in biopsy specimens obtained from the *vastus lateralis* muscle of patients at high risk to develop ICU-acquired muscle wasting and weakness. Accordingly, these patients (n=5) were critically ill, mechanically ventilated with a SOFA score ≥8 on three consecutive days within the first five days after ICU admission. Biopsy specimens were taken at day 15 d after ICU admission. Five age- and gender-matched patients undergoing elective orthopedic surgery permitted muscle biopsies and were used as controls. For more details on the experimental procedure and clinical data, please refer to [1-3]

**Animal model.**

All animal procedures were performed in accordance with the guidelines of the Max-Delbrück Center for Molecular Medicine and the Charité-Universitätsmedizin Berlin, and were approved by the Landesamt für Gesundheit und Soziales (LaGeSo, Berlin, Germany) for the use of laboratory animals (permit numbers G 207/13, G 129/12) and followed the “Principles of Laboratory Animal Care” (NIH publication No. 86-23, revised 1985), as well as the current version of German Law on the Protection of Animals.

To determine the expression of SAA1 receptors in muscle and their regulation in muscle during sepsis we performed cecal ligation puncture (CLP) or sham surgery on 12-week-old male C57Bl6J mice, as recently reported [1, 4, 5]. To investigate the effects of BMS-345541 treatment on muscle in sepsis we used 18-week-old male C57Bl6J mice. Briefly, mice were anesthetized with isoflurane, placed on a heating pad to assure a constant body temperature of 37°C measured by a rectal probe. After shaving and disinfection of the abdominal skin, midline laparotomy was performed, the cecum was exposed and ligated using a non-absorbable surgical suture (Ethicon 6-0). A 21-gauge needle was used to puncture the cecum once, and a small amount of cecum content was extruded. The cecum was then replaced into the abdominal cavity, and the incisions of the peritoneum and skin were closed with two separate layers of surgical sutures (Ethicon 6-0). Sham mice were treated identically except for the ligation and puncture of the cecum. All mice were resuscitated by an i.p. injection of 1 ml sterile and 37°C pre-warmed Ringer’s solution. Mice were sacrificed 96 h after surgery and *gastrocnemius/plantaris*, *tibialis anterior*, *soleus* and the heart were harvested for analysis. The weights of all muscles and the heart were determined, and tibia length was measured for reference purposes. Animals received either vehicle (5 % TWEEN 20 in distilled H_2_O, pH 7.4) or BMS-345541 (30 mg/kg) 30 min prior to, and vehicle or BMS-345541 (15 mg/kg) 6 h after sham or CLP surgery p.o.

**Histological analyses and measurement of myocyte cross-sectional area.**

*Tibialis anterior*, *gastrocnemius/plantaris*, *soleus* and the hearts were obtained from mice that were vehicle or BMS-345541 treated (earlier described) followed by sham or CLP surgery. Muscles were flash frozen in liquid nitrogen with gum tragacanth (Merck, Germany) as cryoprotectant. Histological cross sections with a thickness of 5 µm were cut using the Leica cryotome CM3050S (Leica Microsystems GmbH, Germany) and stained with metachromatic ATPase or Hematoxylin & Eosin (H&E) as described earlier [6, 7]. To analyze the myocyte cross sectional area (MCSA) of type I/slow-twitch (*soleus*) and type II/fast-twitch (for *tibialis anterior*, *gastrocnemius/plantaris* and *soleus*) fibers images were acquired with Leica CTR 6500 HS microscope and the Leica digital camera DFC 425 (Leica Microsystems GmbH, Germany). Image J software 1.51v9 software (Wayne Rasband, National Institutes of Health, USA) was used to measure 100 MSCAs per mouse and condition [4, 8, 2, 9, 10]. MCSA measurements were performed in vehicle (sham n=5, CLP n=4) and BMS-345541 treated mice (sham n=5, CLP n=5). The person who performed measurements was blinded to the specific treatment.

**Myoblast culture and differentiation.**

Cell culture experiments of murine myoblasts (C2C12 cells, ATCC, USA) were performed as previously described [1, 4, 10]. Differentiation of myoblasts to myotubes was induced at confluence by replacing growth medium (Dulbecco's Modified Eagle's medium (DMEM, 1 g/l glucose, Merck, Germany), 10 % fetal bovine serum (FBS, Biochrom GmbH, Germany), supplemented with Penicillin and Streptomycin (Merck, Germany)), by differentiation medium ((DMEM, 1 g/l glucose; Merck, Germany; 2 % FBS, Biochrom GmbH, Germany), supplemented with Penicillin and Streptomycin (Merck, Germany)).

**Immunostaining of C2C12 myotubes.**

C2C12 cells were differentiated on coverslips for 5 d and treated as indicated. Afterwards, cells were fixed with 4 % paraformaldehyde/PBS, permeabilized with 0.2 % Triton-X-100/PBS and blocked with 2 % goat serum/PBS. Myosin heavy chain was detected using anti-MF20 antibody (1:500, R&D Systems, USA) as primary and Alexa flour 488 Goat Anti-Rabbit IgG (H+L) (1:500, Invitrogen) as secondary antibody. DAPI (4',6-Diamidino-2-Phenylindole, Dihydrochloride, 300 mM, Thermo Fisher Scientific, USA) was used to stain for nuclei. Immunostained cells were embedded in ProLong^TM^ Gold antifade mountant (Invitrogen, USA) and analyzed using Leica DM TCS SPE confocal microscope (Leica, Wetzlar, Germany) with Leica software (LCS AF Lite, version 4.0).

**Measurement of myotube diameter.**

Measurements myotube diameter was performed as previously published [10, 4]. In brief, five days differentiated C2C12 myotubes were treated with recombinant human Apo-SAA1 (10 µg/ml, Peprotech, USA), Lipopolysaccharides (1 µg/ml, from Escherichia coli O111:B4, Merck, Germany) or Pam_3_Csk_4_ (50 ng/ml, InvivoGen, UK) in combination with anti-TLR2 antibody (mAb T2.5, 10 µg/ml, Thermo Fisher Scientific, USA), anti-TLR4 antibody (mAb MTS510, 20 µg/ml, BioLegend, USA) or BMS-345541 (5 µM, Abcam, UK) for 72 h. All treatments were repeated after 24 h and 48 h. Light microscope pictures were obtained using the Leica CTR 6500 microscope and the Leica DFC 360 FX digital camera (Leica, Wetzlar, Germany). Per condition, 100 myotubes were analyzed by measuring the diameter of each tube three times with the ImageJ 1.51v9 software (Wayne Rasband, National Institutes of Health USA). The person who performed the measurements was blinded to the specific treatments.

**Quantification of IL6 secreted by myotubes in the cell culture supernatant.**

First, five days differentiated myotubes were treated with SAA1 (1, 10 or 20 µg/ml, as indicated) or vehicle for 9 h. Second, five days differentiated myotubes were pretreated with anti-TLR2 antibody (10 µg/ml, Thermo Fisher Scientific, USA), anti-TLR4 antibody (20 µg/ml BioLegend, USA) or BMS-345541 (25 µM, Abcam, UK) for 1 h followed by 16 h of SAA1 (10 µg/ml, Peprotech, USA) treatment. The cell culture supernatant was collected and the amount of IL6 was measured using the Mouse IL-6 DuoSet ELISA (R&D Systems, DY406, USA) according to the manufactures protocol.

**Protein extraction and Western blot analysis.**

C2C12 cells were differentiated for 5 days and treated with recombinant human Apo-SAA1 (10 µg/ml, Peprotech, USA) or BMS-345541 (5 µM, Abcam, UK), as indicated, for 72 h. Treatments were repeated after 24 h and 48 h. Western blot analysis of protein lysates was performed determine myosin heavy chain 7 (MyHC7) and MuRF1 protein contents. p65 and phospho p65 were detected by Western blot analysis in protein lysates of five days differentiated C2C12 myotubes that were treated for 25 min with recombinant human Apo-SAA1 (10 µg/ml, Peprotech, USA) following 1 h of pretreatment with BMS-345541 (25 µM, Abcam, UK).

Western blot analysis was performed as recently published [4, 10, 8, 9]. Shortly, cells or muscle biopsy samples were lysed in ice-cold extraction buffer (10 mM Tris HCl, pH 7.5, 140 mM NaCl, 1 mM EDTA, 25 % glycerol, 0.5 % sodium dodecyl sulfate (SDS), 0.5 % Nonident P-40, 0.1 mM dithiothreitol, 0.5 mM phenylmethylsulfonyl fluoride, 100 ng/ml protease inhibitor cocktail) and then cleared by centrifugation (4°C, 15 min, 12,000 x g). The Bio-Rad Protein Assay was used to quantitate protein concentration in the supernatant. Isolated proteins were frozen and stored at -80°C until usage. For Western blot analysis 20 μg protein was separated by 10 % SDS polyacrylamide gel electrophoresis (SDS-PAGE) and blotted onto PVDF membranes (0.2 µm, GE Healthcare, UK). Membranes were incubated with specific primary antibodies: anti-glyceraldehyde-3-phosphate dehydrogenase (GAPDH, clone 6C5, Millipore, USA, 1:1000), anti-Skeletal myosin (Slow; clone NOQ7.5.4D, Merck, Germany, 1:1000), anti-NF-kappa B p65 (NF-ĸB p65, clone C-20, Santa Cruz Biotechnology, USA, 1:2000), anti-phospho NF-kappa B p65 (Phospho-NF-κB p65, Serin536, Cell Signaling, UK, 1:500) and anti-Fbx32/Atrogin1 (Abcam, UK, 1:500), and secondary horseradish peroxidase (HRP) conjugated antibodies: anti-mouse IgG (Cell Signaling, UK, 1:20000), anti-rabbit IgG (Cell Signaling, UK, 1:20000), and anti-goat IgG (Abcam, UK, 1:20000). The signal was visualized with SuperSignal™ West Pico Chemiluminescent Substrate (Thermo Fisher Scientific, USA).

**RNA isolation, cDNA synthesis and quantitative real-time-PCR.**

Five days differentiated C2C12 cells were pretreated with anti-TLR2 antibody (mAb T2.5; 10 µg/ml, eBioscience), anti-TLR4 antibody (mAb MTS510; 20 µg/ml, BioLegend, USA) or BMS-345541 (5 µM, Abcam, UK) for 4 h followed by 4 h of stimulation with Apo-SAA1 (10 µg/ml, Peprotech, USA), Lipopolysaccharides (1 µg/ml, from Escherichia coli O111:B4, Merck, Germany) or Pam_3_Csk_4_ (50 ng/ml, InvivoGen, USA).

Total RNA was isolated from cultivated C2C12 cells and skeletal muscle biopsy specimens using TRIzol® Reagent (Invitrogen, USA) according to the manufacturer’s protocol [11, 6, 2]. The SuperScript® First-Strand Synthesis System (Invitrogen, USA) was used for cDNA synthesis of 1 µg of RNA per sample. Quantitative real-time polymerase chain reaction (qRT-PCR) was performed using Power SYBR® Green PCR Master Mix (Merck, Germany) and self-designed primers (for primer sequences, see Additional file 2). TaqMan assays were performed with TaqMan™ Fast Universal PCR Master Mix (2X), no AmpErase™ UNG and TaqMan™ Gene Expression Assays for *TLR2* (Hs00152932_m1), *TLR4* (Hs00152939_m1) and *GAPDH* (Hs02786624_g1; all from Thermo Fisher Scientific, USA). PCR reactions were performed in a Step-One$™$ Plus thermocycler (Applied Biosystems, USA) as described recently using a cDNA standard curve [12, 4, 1, 9, 8, 2, 10]. To correct for potential variances between samples regarding differences in mRNA extraction and reverse transcription efficiency, gene expression was normalized to the expression of the reference *gene glyceraldehyde-3-phosphate dehydrogenase* (*GAPDH*) [12, 4, 1, 9, 8, 2, 10].

**Electrophoretic mobility shift assay.**

H2K/NF-κB oligonucleotide preparation was performed with high-performance liquid chromatography-purified BamHI digested probes (5’-gatcCAGGGCTGGGGATTCCCCATCTCCACAGG-3’ and 5’-gatcCCTGTGGAGATGGGGAATCCCCAGCCCTG-3’). Oligonucleotides were annealed by exposing them to heat (10 min, 95 °C) and slow cooling to room temperature. The final concentration of annealed oligonucleotides was 200 ng/µl. For radioactive labelling of H2K/NF-κB probe the QIAquick Nucleotide Removal Kit (Qiagen, Germany) was used according to manufacturer’s instructions. Annealed and radioactive labelled H2K/NF-κB probe was stored at -20°C until usage. EMSA was performed as previously described [13]. Supershift of NF-κB bands was obtained following preincubation of samples with anti-NF-kappa B p65 antibody (NF-κB p65, clone C-20, Santa Cruz Biotechnology, USA, 1 µg/0.1 ml) for 30 min on ice before EMSA was performed. Following samples were used: five days differentiated C2C12 myotubes pretreated with BMS-345541 (25 µM, Abcam, UK) for 1 h followed by treatment with human recombinant Apo-SAA1 (10 µg/ml, Peprotech, USA) for 25 min.

**Flow cytometry and antibodies**

Spleen cell suspensions were obtained as described [14]. The following antibodies from Biolegend, USA were used: CD4 (Clone: RM 4-5 and GK 1.5, 1:500), CD25 (Clone: PC 61, 1:50), CD69 (Clone: H1.2F3, 1:500), CD11c (Clone: N418, 1:200), CD19 (Clone: 6D5, 1:200), CD3e (Clone: 145-2C11, 1:50) CD8a (Clone 53-6.7, 1:50) and NK 1.1 (Clone: PK136, 1:100). Antibodies against CD11b (Clone: M1/70, 1:200), B220 (Clone: RA3-6B2, 1:50) and Ly6G (Clone 1A8, 1:200) were obtained from BD Bioscience, USA. CD11b (Clone M1/70, 1:50), MHCII (Clone: M5/114.1, 1:200) and isotype control antibodies (Clone: eB149/10H5, 1:200) were from Thermo Fisher Scientific, USA were used. For Foxp3 staining the Foxp3 antibody (Clone: 3G3, 1:10) and the Foxp3 staining set from Miltenyi, Germany were used. Streptavidin-PE-Cy7 (1:200) and 7-AAD viability staining (50 µg/ml) were obtained from Biolegend, USA. The results of the flow cytometry assays were statistically analysed using a one-way ANOVA followed by Dunn’s posttest.

**Supplementary Table 1. Primer pairs for quantitative RT-PCR.**

| Gene | Sequence (5´- 3´) |
| --- | --- |
| Mm_*Gapdh* forward  Mm_*Gapdh* reverse  Mm_*Il6* forward  Mm_*Il6* reverse  Mm_*Tlr2* forward  Mm_*Tlr2* reverse  Mm_*Tlr4* forward  Mm_*Tlr4* reverse  Mm_*Cd36* forward  Mm_*Cd36* reverse  Mm_*P2rx7* forward  Mm_*P2rx7* reverse  Mm_*Vimp* forward  Mm_*Vimp* reverse  Mm_*Scarb1* forward  Mm_*Scarb1* reverse  Mm_*Myh4* forward  Mm_*Myh4* reverse  Mm_*Myh7* forward  Mm_*Myh7* reverse  Mm_*Fbxo30* forward  Mm_*Fbxo30* reverse  Mm_*Fbxo32* forward  Mm_*Fbxo32* reverse  Mm_*Trim63* forward  Mm_*Trim63* reverse | ATG GTG AAG GTC GGT GTG A  AAT CTC CAC TTT GCC ACT GC  CAC TTC ACA AGT CGG AGG CT  TCT GTA TCT CTC TGA AGG ACT CTGACTTACCGAAACCTCAGACAAAGC  AAGACCTGGAGCGGCCAT  CCAAGAACATAGATCTGAGCTTCA  AGAGGTGGTGTAAGCCATGC  TGATGTGCAAAACCCAGATG  AAGACACAGTGTGGTCCTCG  AGGGTGGGGTGACGAAGTTA  CTGCACTTGGCCTTCTGACT  AAATCTGACAAAAAGCCTTTGC  TCCAGGAGCAGGTTCCAC  CGTTGTCATGATCCTCATGGT  ACAGGCTGCTCGGGTCTAT  GGGAACATGAAATTCAAGCAA  ATAGGCAGCCTTGTCAGCAA  CGCATCAAGGAGCTCACC  CTGCAGCCGCAGTAGGTT  GAG AAG CCA GGG TTT GAG C  TCA TAC AGT GTG AGT GCT GCT G  AGT GAG GAC CGG CTA CTG TG  GAT CAA ACG CTT GCG AAT CT  TGACATCTACAAGCAGGAGTGC  TCGTCTTCGTGTTCCTTGC |

*Gapdh* indicates Glyceraldehyde 3-phosphate dehydrogenase; *IL6*, interleukin 6; *Tlr2*, toll-like receptor 2; *Tlr4*, toll-like receptor 4; *Cd36*, cluster of differentiation 36; *P2rx7*, P2X purinoceptor 7; *Vimp*, VCP interacting membrane selenoprotein; *Scarb1*, scavenger receptor class b member 1; *Myh4*, myosin heavy chain 4; *Myh7*, myosin heavy chain 7; *Fbxo30*, F-box protein 30; *Fbxo32*, F-box protein 32; *Trim63*, tripartite motif containing 63.

**Supplementary Figure 1. BMS-345541 treated mice are protected from sepsis-induced cardiac atrophy.** 18-week-old male WT mice were either sham or CLP operated as indicated. Per surgical group mice were divided into two groups each which than received either vehicle (5 % TWEEN 20 in distilled H_2_O, pH 7.4) or BMS-345541 30 min prior to and 6 h after surgery p.o.; experimental groups are as following: sham + vehicle (n = 6), CLP + vehicle (n = 4), sham + BMS-345541 (n = 6), and CLP + BMS-345531 (n = 11). After 96 h the mice were sacrificed. **A** Hematoxylin and eosin (H&E) staining of histological cross sections from the heart.

**Supplementary Figure 2. No significant influence of BMS-345541 on sepsis-induced splenic weight gain or the number of splenocytes**. 18-week-old C57Bl6J mice received either vehicle (5 % TWEEN 20 in distilled H2O, pH 7.4) or BMS-345541 (30 mg/kg) by oral gavage 30 min prior sham (-) or CLP (+) surgery. A second dose of vehicle of BMS-345541 (15 mg/kg) was administered 6 h after surgery. Experimental groups are as following: sham + vehicle (n = 10), CLP + vehicle (n = 4), sham + BMS-345541 (n = 10), and CLP + BMS-345531 (n = 11). After 96 h mice were sacrificed. **A** Relative weight of the spleen normalized to tibia length. **B** Total spleen cell number was quantified by fluorescence-activated cell scanning (FACS). Data are presented as scatter plot and the median is indicated. *p<0.05, ***p<0.001

**Supplementary Figure 3. No significant influence of BMS-345541 on the numbers of dendritic cells, B cells, natural killer cells or neutrophils in the spleen of septic mice**. Experimental procedure and groups are the same as in the additional figure 1. **A** Number of CD11c^hi+^/MHCII^+^ dendritic cells in spleen, and percentage of dendritic cells among splenocytes. **B** Number of CD19^+^/B220^+^ B cells in the spleen, and percentage of B cells among splenocytes. **C** Number of NK1.1^+^ natural killer cells in the spleen and percentage of natural killer cells among splenocytes. **D** Number of Ly6G^+^ neutrophils in spleen and percentage of neutrophil cells among splenocytes. Data are presented as scatter plot and the median is indicated. *p<0.05, **p<0.01, ***p<0.001

**Supplementary Figure 4. No influence of BMS-345541 on the numbers of T cells, T helper cells or cytotoxic T cells in the spleen of septic mice**. Experimental procedure and groups are the same as in the additional figure 1. **A** Number of CD3^+^ T cells in the spleen and percentage of T cells among splenocytes. **B** Number of CD4^+^ T helper cells in the spleen and percentage of T helper cells among splenocytes **C** Number of CD8^+^ cytotoxic T cells in the spleen and percentage of cytotoxic cells among splenocytes. Data are presented as scatter plot and the median is indicated. *p<0.05, **p<0.01.

**Supplementary Figure 5. No significant influence of BMS-345541 on effector- or regulatory T cell numbers or on their activation in the spleen of septic mice.** Experimental procedure and groups are the same as in the additional figure 1. **A** Total splenocyte counts of Foxp3^+^, CD25^+^/CD4^+^ regulatory T cell and Foxp3^-^, CD25^+^ effector T cells in the spleen. **B** Percentage of Foxp3^+^, CD25^+^/CD4^+^ regulatory T cell and Foxp3^-^, CD25^+^ effector T cells of total splenocytes. Data are presented as scatter plot and the median is indicated. **C** Numbers of activated (CD69^+^) regulatory T cells and CD69/effector T cells in spleen. Data are presented as scatter plot and the median is indicated. *p<0.05, **p<0.01, ***p<0.001.

**Supplementary References**

1. Langhans C, Weber-Carstens S, Schmidt F, Hamati J, Kny M, Zhu X, et al. Inflammation-induced acute phase response in skeletal muscle and critical illness myopathy. PloS one. 2014;9(3):e92048.

2. Wollersheim T, Woehlecke J, Krebs M, Hamati J, Lodka D, Luther-Schroeder A, et al. Dynamics of myosin degradation in intensive care unit-acquired weakness during severe critical illness. Intensive care medicine. 2014;40(4):528-38.

3. Wollersheim T, Grunow JJ, Carbon NM, Haas K, Malleike J, Ramme SF, et al. Muscle wasting and function after muscle activation and early protocol-based physiotherapy: an explorative trial. Journal of cachexia, sarcopenia and muscle. 2019.

4. Huang N, Kny M, Riediger F, Busch K, Schmidt S, Luft FC, et al. Deletion of Nlrp3 protects from inflammation-induced skeletal muscle atrophy. Intensive care medicine experimental. 2017;5(1):3.

5. Rittirsch D, Huber-Lang MS, Flierl MA, Ward PA. Immunodesign of experimental sepsis by cecal ligation and puncture. Nat Protoc. 2009;4(1):31-6.

6. Fielitz J, Kim M-S, Shelton JM, Latif S, Spencer JA, Glass DJ, et al. Myosin accumulation and striated muscle myopathy result from the loss of muscle RING finger 1 and 3. The Journal of clinical investigation. 2007;117(9):2486-95.

7. Kim MS, Fielitz J, McAnally J, Shelton JM, Lemon DD, McKinsey TA, et al. Protein kinase D1 stimulates MEF2 activity in skeletal muscle and enhances muscle performance. Mol Cell Biol. 2008;28(11):3600-9.

8. Schmidt F, Kny M, Zhu X, Wollersheim T, Persicke K, Langhans C, et al. The E3 ubiquitin ligase TRIM62 and inflammation-induced skeletal muscle atrophy. Critical care (London, England). 2014;18(5):545.

9. Lodka D, Pahuja A, Geers-Knorr C, Scheibe RJ, Nowak M, Hamati J, et al. Muscle RING-finger 2 and 3 maintain striated-muscle structure and function. Journal of cachexia, sarcopenia and muscle. 2016;7(2):165-80.

10. Zhu X, Kny M, Schmidt F, Hahn A, Wollersheim T, Kleber C, et al. Secreted Frizzled-Related Protein 2 and Inflammation-Induced Skeletal Muscle Atrophy. Crit Care Med. 2017;45(2):e169-e83.

11. Fielitz J, Kim MS, Shelton JM, Qi X, Hill JA, Richardson JA, et al. Requirement of protein kinase D1 for pathological cardiac remodeling. Proc Natl Acad Sci U S A. 2008;105(8):3059-63.

12. Du Bois P, Pablo Tortola C, Lodka D, Kny M, Schmidt F, Song K, et al. Angiotensin II Induces Skeletal Muscle Atrophy by Activating TFEB-Mediated MuRF1 Expression. Circ Res. 2015;117(5):424-36.

13. Stilmann M, Hinz M, Arslan SC, Zimmer A, Schreiber V, Scheidereit C. A nuclear poly(ADP-ribose)-dependent signalosome confers DNA damage-induced IkappaB kinase activation. Mol Cell. 2009;36(3):365-78.

14. Busse M, Traeger T, Potschke C, Billing A, Dummer A, Friebe E, et al. Detrimental role for CD4+ T lymphocytes in murine diffuse peritonitis due to inhibition of local bacterial elimination. Gut. 2008;57(2):188-95.
